# The Dangers of Ether

**Published:** 1877-12

**Authors:** 


### THE DANGERS OF ETHER.

It has always seemed to us the height of folly to declare there could be no danger in any anesthetic. The lesson taught by a late death from nitrous oxide has, it is to be hoped, been well learned, and we shall in future hear less of the absolute safety of any agent capable of depriving a person of all sensation. Some cases in which ether has been followed by alarming symptoms have lately been recorded. They have been termed syncope, but the word is not appropriate, as the heart continued to beat after respiration ceased. This is what should have been anticipated. When death is produced by ether, the animal's heart continues to beat long after the arrest of respiration. The pulse is quickened by ether, and maintains its force through a long state of anæthesia. In these facts lies the saftey of ether. But it should never be forgotten that there is danger at a certain stage, and the danger is from the side of the respiration, which at length ceases.

Stertorous breathing proceeds from paresis of the muscles of the palate, and should lead to the ether being suspended. So respiration, growing more and more shallow and less frequent, is a warning, and should not be overlooked. It is very rare that the heart fails—perhaps never. Pallor is rare, too, and should excite attention if it occur. But, we repeat, the danger of ether is from the side of respiration, that of choloform from the heart, and this fact goes far to explain their relative safety. In choloform narcosis the danger is much more sudden. Ether gives warning.—*The Doctor, London.*

